# Differential expression of components of the CGRP-receptor family in human coronary and human middle meningeal arteries: functional implications

**DOI:** 10.1186/s10194-024-01863-7

**Published:** 2024-10-10

**Authors:** Tessa de Vries, Dennis Schutter, Antoon van den Bogaerdt, Arnaud Vincent, Ruben Dammers, A. H. Jan Danser, Antoinette MaassenVanDenBrink

**Affiliations:** 1 Division of Vascular Medicine and Pharmacology, Department of Internal Medicine, Erasmus MC, University Medical Center Rotterdam, PO Box 2040, Rotterdam, 3000 CA The Netherlands; 2 Heart Valve Department, ETB-BISLIFE, Beverwijk, The Netherlands; 3 Department of Neurosurgery, Erasmus MC, University Medical Center Rotterdam, Rotterdam, The Netherlands

**Keywords:** Adrenomedullin, AM_22-52_, CGRP, Human arteries, Olcegepant, qPCR, Receptor expression, Vasodilation

## Abstract

**Background:**

Different responses in human coronary arteries (HCA) and human middle meningeal arteries (HMMA) were observed for some of the novel CGRP receptor antagonists, the gepants, for inhibiting CGRP-induced relaxation. These differences could be explained by the presence of different receptor populations in the two vascular beds. Here, we aim to elucidate which receptors are involved in the relaxation to calcitonin gene-related peptide (CGRP), adrenomedullin (AM) and adrenomedullin 2 (AM2) in HCA and HMMA.

**Methods:**

RNA was isolated from homogenized human arteries (23 HCAs; 12 F, 11 M, age 50 ± 3 years and 26 HMMAs; 14 F, 12 M, age 51 ± 3 years) and qPCR was performed for different receptor subunits. Additionally, relaxation responses to CGRP, AM or AM2 of the human arteries were quantified using a Mulvany myograph system, in the presence or absence of the adrenomedullin 1 receptor antagonist AM_22-52_ and/or olcegepant.

**Results:**

Calcitonin-like receptor (CLR) mRNA was expressed equally in both vascular beds, while calcitonin receptor (CTR) and receptor activity-modifying protein 3 (RAMP3) expression was low and could not be detected in all samples. RAMP1 expression was similar in HCA and HMMA, while RAMP2 expression was higher in HMMA. Moreover, receptor component protein (RCP) expression was higher in HMMA than in HCA. Functional experiments showed that olcegepant inhibits relaxation to all three agonists in both vascular beds. In HCA, antagonist AM_22-52_ did not inhibit relaxation to any of the agonists, while a trend for blocking relaxation to AM and AM2 could be observed in HMMA.

**Conclusion:**

Based on the combined results from receptor subunit mRNA expression and the functional responses in both vascular tissues, relaxation of HCA is mainly mediated via the canonical CGRP receptor (CLR-RAMP1), while relaxation of HMMA can be mediated via both the canonical CGRP receptor and the adrenomedullin 1 receptor (CLR-RAMP2). Future research should investigate whether RAMP2 predominance over RAMP1 in the meningeal vasculature results in altered migraine susceptibility or in a different response to anti-migraine medication in these patients. Moreover, the exact role of RCP in CGRP receptor signalling should be elucidated in future research.

**Supplementary Information:**

The online version contains supplementary material available at 10.1186/s10194-024-01863-7.

## Background

Calcitonin gene-related peptide (CGRP) is a potent vasodilator that is involved in the pathophysiology of migraine. Infusion of CGRP can induce migraine-like headaches in migraine patients [1, 2] and activation of the trigeminovascular system during a migraine attack results in the release of neuropeptides, including CGRP [3, 4]. Therefore, novel anti-migraine drugs target the CGRP receptor, or the CGRP peptide itself. Two classes of CGRP(-receptor) targeting medication have been developed, i.e. the small molecule CGRP receptor antagonists (gepants) and the monoclonal antibodies targeting CGRP (eptinezumab, fremanezumab, galcanezumab) or the CGRP receptor (erenumab), and both have been shown to be effective in the treatment of migraine [5, 6].

**Fig. 1:**
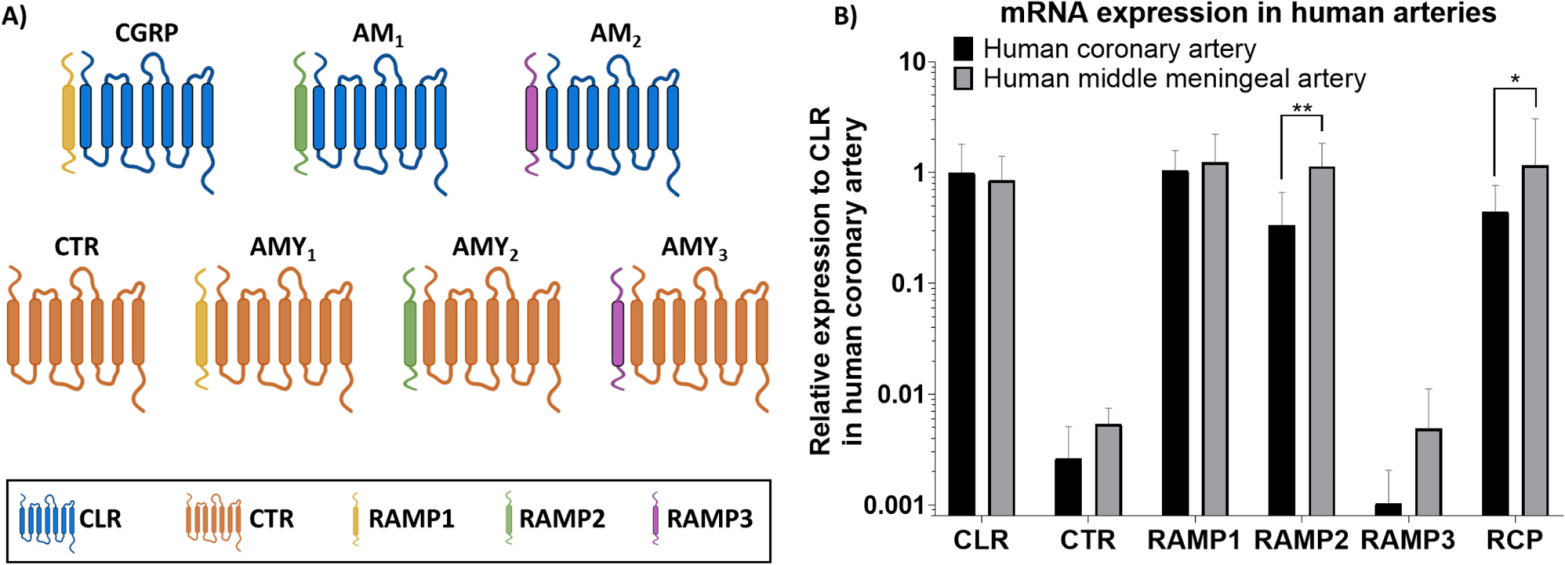
mRNA expression of receptor subunits in the human coronary artery and human middle meningeal artery. **A**) Overview of the different receptors within the calcitonin gene-related peptide family and the transmembrane proteins that constitute the receptor. **B**) Expression of CLR, CTR, RAMP1, RAMP2, RAMP3 and RCP in human coronary artery (*n* = 23) and human middle meningeal artery (*n* = 26). Data are expressed as mean ± SD. *p < 0.05, **p < 0.01

CGRP is part of a family of related peptides that have similar characteristics and can cross-activate each other’s receptors [7]. The canonical CGRP receptor consists of the seven transmembrane G protein coupled receptor calcitonin-like receptor (CLR) coupled to a single transmembrane protein called receptor activity modifying protein 1 (RAMP1). In addition, the accessory protein receptor component protein (RCP) is located on the intracellular side of the receptor [8]. When CLR is coupled to a different receptor activity modifying protein, it can form the adrenomedullin 1 receptor (CLR-RAMP2) or the adrenomedullin 2 receptor (CLR-RAMP3) (Fig. 1A). Additionally, instead of the CLR subunit, the calcitonin receptor (CTR) can be coupled to either of the RAMP subunits to form the amylin 1 (CTR-RAMP1), amylin 2 (CTR-RAMP2) and amylin 3 (CTR-RAMP3) receptor, or it can function as a receptor on its own. The peptides adrenomedullin, adrenomedullin 2, calcitonin and amylin are also part of the CGRP peptide family and can target these receptors with varying potencies. CGRP is the most potent agonist at the canonical CGRP receptor, but this receptor can also be activated by adrenomedullin and adrenomedullin 2 [7]. In contrast, adrenomedullin and adrenomedullin 2 are more potent at activating the adrenomedullin 1 receptor than CGRP.

Our previous research has shown that the small molecule CGRP receptor antagonists, the gepants, behave differently in different vascular tissues [9] and it was hypothesized that these differences arise from differential receptor expression in the vascular beds. This could have consequences for the effect of this novel anti-migraine medication throughout the human vasculature, by affecting each artery differently. Importantly, in case of cardiac or cerebral ischemia, CGRP can induce vasodilation, thereby protecting the affected tissue by enhancing blood flow [10, 11]. Therefore, blocking CGRP signalling could induce adverse effects in case of ischemia [12]. Indeed, different gepants have been shown to aggravate cerebral ischemia in mice after middle cerebral artery occlusion [13]. It is important to investigate via which receptors CGRP induces vasodilation in the different vascular tissues and which arteries are mainly affected by CGRP receptor blockade. Therefore, the current study aims to investigate the mRNA expression of all aforementioned receptor subunits of the CGRP receptor family in human coronary arteries and human middle meningeal arteries to determine which receptors are expressed in the different arteries. Moreover, functional responses to CGRP, adrenomedullin and adrenomedullin 2 are studied in the presence of the CGRP receptor antagonist olcegepant and the adrenomedullin 1 receptor antagonist adrenomedullin-(22–52) (AM_22-52_) to determine whether the responses by these agonists can be blocked using specific inhibitors and are thus mediated via these receptors.

## Methods

Human coronary arteries were isolated from hearts of Dutch post-mortem heart valve donors, which were provided by ETB-BISLIFE (Heart Valve Department, Beverwijk, The Netherlands) following removal of the aortic and pulmonary valve for homograft valve transplantation. Donor screening and acceptance was performed by the Dutch Transplant Foundation (Leiden, The Netherlands) and all donors gave permission for research. Immediately after circulatory arrest, the hearts were harvested and stored at 4 °C in a sterile organ protecting solution and were brought to the laboratory within 24 h of death for valve isolation. Subsequently, coronary arteries with an inner diameter between 300 μm and 2 mm were isolated. For qPCR experiments, arteries were snap frozen and stored at -80 °C until RNA isolation. For functional experiments, arteries were stored in carbogenated (95% O_2_ and 5% CO_2_) Krebs solution (118 mM NaCl, 4.7 mM KCl, 2.5 mM CaCl_2_, 1.2 mM MgSO_4_, 1.2 mM KH_2_PO_4_, 25 mM NaHCO_3_ and 8.3 mM glucose, pH = 7.4) at 4 °C until the start of the Mulvany myograph experiments.

Human middle meningeal arteries were obtained from patients undergoing neurosurgical procedures at the Erasmus Medical Center, Rotterdam, The Netherlands. Middle meningeal arteries were stored in Medium 199 (Capricorn Scientific) and transported to the lab immediately. Subsequently, surrounding tissue was removed and the artery was snap frozen and stored at -80 °C for qPCR experiments or stored in a cold carbogenated (95% O_2_ and 5% CO_2_) Krebs solution [14] (119 mM NaCl, 4.7 mM KCl, 1.25 mM CaCl_2_, 1.2 mM MgSO_4_, 1.2 mM KH_2_PO_4_, 25 mM NaHCO_3_ and 11.1 mM glucose, pH = 7.4) at 4 °C until the start of the myograph experiments.

For qPCR experiments, snap frozen arteries were homogenized manually and lysed using TRIzol. RNA was isolated by phase separation using chloroform, followed by precipitation using isopropyl alcohol. The RNA pellet was washed with ethanol and dissolved in RNase-free water. Nucleic acid concentrations were measured using NanoDrop and samples were stored at -80 °C until further use. Samples with a 260/280 or 260/230 value below 1.85 underwent an additional overnight precipitation step using ethanol and sodium acetate for further purification. Next, cDNA synthesis and genomic DNA elimination was executed using the Maxima H Minus First Strand cDNA Synthesis Kit (ThermoFisher Scientific) according to the manufacturer’s instructions. The qPCR measurements were performed using a CFX384 Thermal Cycler (Bio-Rad Laboratories) in a 10 µl reaction volume containing SYBR Green PCR Select Master Mix (Applied Biosystems), cDNA and primers (Table S1). Primers were selected when a single peak was visible during a melt curve and when the standard curve with serially diluted cDNA showed an efficiency of above 90%. All qPCR reactions were performed in duplicate, starting with 2 min at 50 °C, followed by 2 min at 95 °C and subsequently 40 cycles of 15 s at 95 °C followed by 1 min at 60 °C. Three different reference genes were used, i.e. β-actin, GAPDH and HPRT1 for normalization of the data. Data are expressed as the relative expression of each gene of interest (CLR, CTR, RAMP1, RAMP2, RAMP3, RCP) to CLR in human coronary arteries, corrected for the expression of the three reference genes. A mixed-effects analysis was performed to study the differences in mRNA expression followed by a Bonferroni’s multiple comparisons test to look at differences in receptor subunit expression between human coronary arteries and human middle meningeal arteries.

For functional experiments, human arteries were cut into 2 mm segments and mounted in Mulvany myograph organ baths (Danish Myo Technology, Aarhus, Denmark), using Ø 40-µm stainless-steel wires. Organ baths were filled with carbogenated Krebs solution at 37 °C. Vessel segments were left to equilibrate. Next, the segments were stretched to a tension normalized to 0.9 times the estimated diameter at 100 mmHg transmural pressure [15]. Data were recorded using LabChart data acquisition software (AD instruments Ltd, Oxford, UK). First, all segments were exposed to 30 mM KCl, followed by 100 mM KCl. After washing twice, the vessels were pre-contracted using 30 mM KCl, and exposed to increasing concentrations (0.01 nM – 1 µM, in half logarithmic steps) of human α-CGRP (Polypeptide Group, Baar, Switzerland), adrenomedullin (Bachem, Switzerland) or adrenomedullin 2 (Bachem, Switzerland). Segments were incubated with or without 1 µM olcegepant (MedChemExpress, Monmouth Junction, USA), 1 µM AM_22-52_ (Bachem, Switzerland) or 1 µM olcegepant + 1 µM AM_22-52_ in a paired, parallel experimental setup. Incubation with the antagonist started 30 min before the first concentration of agonist was added to study the inhibition of relaxation. After the concentration-response curve to the different agonists, segments were washed and precontracted using U46619 (10–100 nM) followed by the endothelium-dependent vasodilator substance P (10–100 nM) to verify endothelial integrity. For data analysis of the functional experiments, concentration-response curves with a sigmoidal shape were obtained and analysed using Prism 8 (GraphPad Software, San Diego, CA, USA). Non-linear regression analysis was used to determine the pEC_50_ values. For fitting of the curves, the maximum relaxation response was assumed to be ≤ 100%, similar among all curves with the same agonist, and unaffected by incubation with an antagonist. Next, estimated pEC_50_ values were compared using a mixed effects analysis, and if significant followed by a Dunnett’s multiple comparisons test to compare the experiments with the different antagonists to the control. For all statistical analyses, *p* < 0.05 was considered significant.

## Results

The mRNA expression of components of the CGRP receptor family was assessed in 23 human isolated coronary arteries (12 female, 11 male, age 50 ± 3 years) and 26 human middle meningeal arteries (14 female, 12 male, age 51 ± 3 years; Fig. 1A). The expression of CLR and RAMP1 subunits, which are both part of the canonical CGRP receptor, was similar in both vascular beds. However, mRNA expression of RCP, which is the third subunit of the canonical CGRP receptor, did differ between the two tissues, with a significantly higher expression in human middle meningeal arteries compared to human coronary arteries (*p* = 0.0186). The mRNA levels of CTR and RAMP3 were low and could not consistently be detected in all samples. Interestingly, the expression of RAMP2 was significantly different between the two vascular tissues, with an approximately 3.4 times higher expression in the human middle meningeal arteries (*p* = 0.0055). Together with CLR, RAMP2 forms the adrenomedullin 1 receptor.

**Fig. 2:**
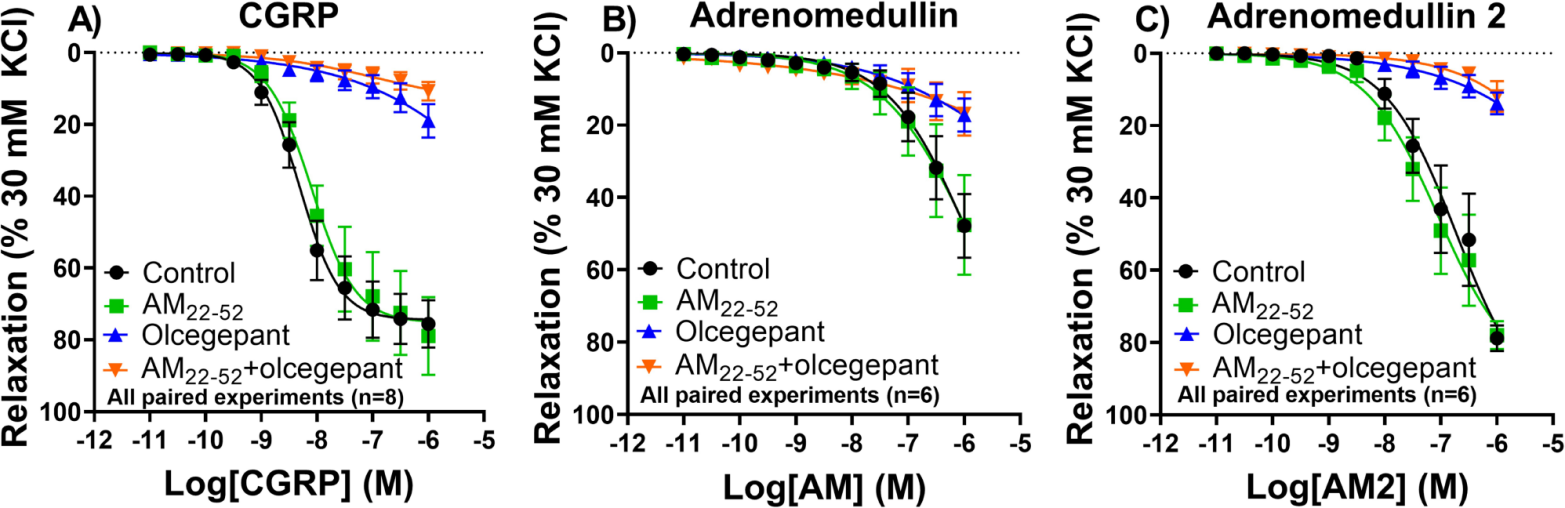
Concentration-response curve to CGRP, adrenomedullin or adrenomedullin 2 in the human coronary artery. Experiments were performed in the presence or absence of CGRP receptor antagonist olcegepant (1 µM) and adrenomedullin receptor antagonist AM_22 − 52_ (1 µM). (**A**) Relaxation to CGRP (*n* = 8). (**B**) Relaxation to adrenomedullin (*n* = 6). (**C**) Relaxation to adrenomedullin 2 (*n* = 6)

To determine whether this differential receptor mRNA expression in the human coronary arteries and human middle meningeal arteries has any functional consequences, concentration-response curves to CGRP, adrenomedullin and adrenomedullin 2 were constructed in both vascular beds. In human coronary arteries (*n* = 9, 6 female, 3 male, age 54 ± 7 years), CGRP could induce the most potent relaxation (pEC_50_ 8.17 ± 0.18) followed by adrenomedullin 2 (pEC_50_ 6.98 ± 0.26) and adrenomedullin (pEC_50_ 6.27 ± 0.27). The endothelial integrity of the vessel segments was assessed by relaxation to the endothelium-dependent vasodilator substance P, with an average of 69 ± 3% relaxation of the precontraction to U46619 in human coronary arteries. No differences could be observed between the segments after incubation with the different antagonists, or between experiments with either of the three agonists, and endothelial quality did not correlate with a larger response to any of the agonists. The relaxation to all three agonists could be inhibited by the CGRP receptor antagonist olcegepant, while the adrenomedullin 1 receptor antagonist AM_22-52_ did not alter the concentration-response curves (Fig. 2; Table 1). In addition, AM_22-52_ did not induce an additional effect on top of olcegepant for either of the three agonists.

**Table 1:** Potency of CGRP, adrenomedullin and adrenomedullin 2 in human coronary artery in the presence of vehicle, olcegepant and/or AM_22 − 52_. The pEC_50_ values of the conditions with the different antagonists are compared with the control pEC_50_. *p < 0.05, **p < 0.01, ***p < 0.001. AM: adrenomedullin, AM2; adrenomedullin 2

	Control	AM_22 − 52_		Olcegepant		AM_22 − 52_ + olcegepant	
	pEC_50_ ± SEM	pEC_50_ ± SEM	sign.	pEC_50_ ± SEM	sign.	pEC_50_ ± SEM	sign.
CGRP	8.17 ± 0.18	7.84 ± 0.27	ns	4.63 ± 0.50	**	4.20 ± 0.33	***
AM	6.27 ± 0.27	6.23 ± 0.26	ns	4.63 ± 0.45	**	4.24 ± 0.69	*
AM2	6.98 ± 0.26	7.14 ± 0.34	ns	4.48 ± 0.40	**	4.32 ± 0.45	*

**Fig. 3:**
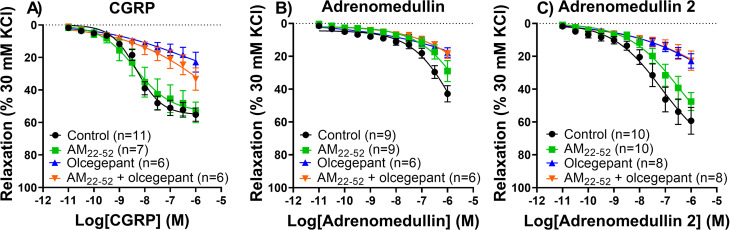
Concentration-response curve to CGRP, adrenomedullin or adrenomedullin 2 in the human middle meningeal artery. Experiments were performed in the presence or absence of CGRP receptor antagonist olcegepant (1 µM) and adrenomedullin receptor antagonist AM_22-52_ (1 µM). (**A**) Relaxation to CGRP (*n* = 6–11). (**B**) Relaxation to adrenomedullin (*n* = 6–9). (**C**) Relaxation to adrenomedullin 2 (*n* = 8–10)

Similar to the human coronary arteries, CGRP was the most potent vasodilator (pEC_50_ 8.16 ± 0.14) in human middle meningeal arteries (*n* = 13, 9 female and 4 male, age 57 ± 12 years), followed by adrenomedullin 2 (pEC_50_ 6.90 ± 0.24) and adrenomedullin (pEC_50_ 5.60 ± 0.23). The endothelial integrity of the vessel segments in human middle meningeal arteries (average relaxation of 55 ± 3% of precontraction with U46619) did not differ between the segments after incubation with the different antagonists, or between experiments with either of the three agonists, and endothelial quality did not correlate with a larger response to an agonist. The relaxation to CGRP (Fig. 3A), adrenomedullin (Fig. 3B) and adrenomedullin 2 (Fig. 3C) could be blocked using olcegepant (Table 2). AM_22-52_ on its own or on top of olcegepant did not attenuate the relaxation to CGRP. The response to adrenomedullin and adrenomedullin 2 was not significantly affected by the antagonist AM_22-52_, although a trend for inhibition could be observed with a 0.82 and 0.68 log unit shift compared to the control curve, respectively (Fig. 3B and C; Table 2).

**Table 2:** Potency of CGRP, adrenomedullin and adrenomedullin 2 in human middle meningeal artery in the presence of vehicle, olcegepant and/or AM_22 − 52_. The pEC_50_ values of the conditions with the different antagonists are compared with the control pEC_50_. *p < 0.05, **p < 0.01. AM: adrenomedullin, AM2; adrenomedullin 2

	Control	AM_22 − 52_		Olcegepant		AM_22 − 52_ + olcegepant	
	pEC_50_ ± SEM	pEC_50_ ± SEM	sign.	pEC_50_ ± SEM	sign.	pEC_50_ ± SEM	sign.
CGRP	8.16 ± 0.14	8.03 ± 0.39	ns	4.32 ± 0.78	**	5.34 ± 0.61	*
AM	5.60 ± 0.23	4.78 ± 0.39	ns	3.59 ± 0.20	**	3.67 ± 0.50	*
AM2	6.90 ± 0.24	6.22 ± 0.48	ns	3.06 ± 1.08	*	4.41 ± 0.69	**

Considering the similar expression of CLR in the two vascular tissues, the expression of RAMP1 versus RAMP2 could determine whether the canonical CGRP receptor or the adrenomedullin 1 receptor is the predominant receptor in the specific vascular bed. Therefore, the relative expression of RAMP1 and RAMP2 per tissue donor was examined in more detail (Figure S1A). For all human coronary arteries, the expression of RAMP1 was higher than or similar to the expression of RAMP2. However, in human middle meningeal arteries, a mixed pattern could be observed, with some tissues showing a higher RAMP2 expression while others have higher RAMP1 expression. In addition, two distinct populations were visible for RAMP2 expression in human middle meningeal artery (Figure S1B), supported by a normality test confirming a non-normal distribution (Shapiro-Wilk test *p* = 0.0392), while the data for RAMP2 in human coronary artery or the data for RAMP1 in both vascular tissues passed the normality test. Based on the two distinct populations with high or low RAMP2 expression, possible differences in functional responses are hypothesised. Indeed, for the relaxation to adrenomedullin and adrenomedullin 2, AM_22-52_ was able to block the relaxation responses in some of the tissues. For adrenomedullin, four out of nine donors showed a clear inhibition with AM_22-52_ (Figure S1C), defined as a shift of ≥ 1 log units (control pEC_50_ 5.65 ± 0.25 and AM_22-52_ pEC_50_ 3.82 ± 0.52, *n* = 4). For adrenomedullin 2, this was the case for four out of ten donors (control pEC_50_ 7.15 ± 0.46 and AM_22-52_ pEC_50_ 5.05 ± 0.88, *n* = 4; Figure S1D). For the other donors, no clear effect of AM_22-52_ could be observed (Figure S1E, S1F).

## Discussion

The current study shows that the expression of the canonical CGRP receptor (CLR-RAMP1) is similar in human coronary arteries and human middle meningeal arteries, while the expression of the adrenomedullin 1 receptor (CLR-RAMP2) differs, with a higher expression in human middle meningeal arteries. The current expression data matches largely with the expression as reported in the Human Protein Atlas [16] for smooth muscle tissue normalized expression (nTPM), with the highest expression of β-actin, followed by GAPDH, RAMP1 and RAMP2, which is exactly the order of expression in human middle meningeal arteries and similar to human coronary arteries except for the lower expression of RAMP2 in this tissue. Indeed, CTR expression is reported to be absent or low in this tissue, again matching our data. It should be noted that our isolated arteries do not exclusively consist of smooth muscle tissue, but also connective tissue and endothelial cells. However, the arteries consist of a thick layer of smooth muscle tissue and only a monolayer of endothelial cells, suggesting that the main contribution to the RNA content in our homogenized arteries is from smooth muscle cells. Further research is needed to determine the relation between mRNA expression and protein expression of all receptor subunits in the vasculature. However, due to the scarcity of the human arteries and the limited amount of tissue obtained per patient, it was not possible to assess both protein expression and mRNA expression within the same tissue. In line with this, mRNA expression and functional responses could not be measured in tissue of the same donor. Therefore, we cannot draw conclusions on whether the functional responses match the expression data in an individual donor. Furthermore, we only have information from the total homogenized arteries and cannot separate the results per cell type (i.e. endothelial cells or smooth muscle cells), or per subcellular compartment. Further research is necessary to determine where exactly the receptor subunits are expressed, and what the physiological relevance of this expression is likely to be.

The findings of the current study in coronary arteries are in accordance with a previous studies in both human and porcine coronary arteries in which mRNA of subunits of the canonical CGRP receptor and the adrenomedullin 1 receptor was detected, while CTR and RAMP3 could not be detected porcine coronary arteries [17, 18]. In addition, RAMP1, RAMP2, RAMP3 and CLR expression have been previously detected in human middle meningeal arteries [19]. The current study offers a direct comparison between these two vascular tissues and allows quantification of expression of the different subunits. Interestingly, in contrast to the vasculature, expression of CTR was previously detected in the human trigeminal ganglion and dorsal root ganglion, where it is likely involved in pain transmission [20, 21].

Interestingly, in depth analysis of the expression data shows two types of expression patterns in human middle meningeal arteries, with some patients having higher RAMP1 expression and others having higher RAMP2 expression. Moreover, our functional results in human middle meningeal arteries show the same two patient groups, of which some respond clearly to the adrenomedullin receptor antagonist AM_22-52_, while others do not. AM_22-52_ is the truncated version of adrenomedullin and is an antagonist at the adrenomedullin 1 receptor, while at a lower potency it can also antagonize the adrenomedullin 2 receptor [22]. The fact that both our functional results and the mRNA expression data can be divided in two populations could suggest that the mRNA expression of RAMP1 and RAMP2 reflects the protein expression in these tissues. In contrast, the adrenomedullin 1 receptor antagonist AM_22-52_ had no effect in human coronary arteries, in which RAMP1 expression is predominant. The absence of antagonistic effects of AM_22-52_ in coronary arteries has been described previously [17, 18].

The novel anti-migraine drugs targeting the canonical CGRP receptor (i.e. gepants and the monoclonal antibody erenumab) are designed to target the hydrophobic pocket between CLR and RAMP1, thereby preventing direct binding of CGRP to the receptor [23, 24]. Gepants and erenumab have a high affinity for the CGRP receptor, and no or only low affinity for the adrenomedullin 1 receptor (CLR-RAMP2) or the adrenomedullin 2 receptor (CLR-RAMP3) [22, 25–28]. Therefore, treatment response could be affected by the exact receptor expression in HMMA, which could serve as a direct target for antimigraine medication, and as a proxy for what is happening in the trigeminovascular system, an important structure involved in the pathophysiology of migraine [29]. Interestingly, the observations in the current study showing two types of expression patterns of RAMP1 and RAMP2 in HMMA donors, could suggest that also two types of (migraine) patients exist, who possibly respond differently to treatment. If in some patients the RAMP2 expression in the human middle meningeal artery is higher than RAMP1, CGRP signalling could continue via the adrenomedullin 1 receptor even if the CGRP receptor is blocked, resulting in less effective treatment with the CGRP receptor antagonists. Future studies should determine whether receptor subunit expression differs between migraine patients and healthy controls. Unfortunately, the migraine status of the patients included in the current study, or their medication use, is unknown because of ethical regulations, anonymizing our donors.

One of the interesting findings of the current study is the differential expression of RCP, with higher expression in the human middle meningeal arteries compared to human coronary arteries. The exact role of this receptor subunit has been debated [8]. RCP is located on the intracellular side of the G protein coupled receptor CLR, and was shown to co-immunoprecipitate with both the canonical CGRP receptor and the adrenomedullin 1 receptor [30]. Therefore, since both receptors are abundant in human middle meningeal arteries, while the canonical CGRP receptor is predominant in human coronary arteries, the increased expression of RCP in human middle meningeal arteries could be because it forms a functional unit of both receptors present in the meningeal vasculature. It has previously been shown that loss of RCP does not affect receptor density or receptor binding, while it does affect intracellular cAMP production [30, 31], suggesting a role for intracellular signalling. Interestingly, depletion of RCP had a larger effect on cAMP levels after stimulation with CGRP compared with stimulation with adrenomedullin [30]. It should be noted that RCP protein levels were poorly correlated with RCP mRNA expression in mouse uterus during pregnancy [32], and it is suggested that RCP mRNA levels might not accurately predict RCP function.

## Conclusion and future perspectives

CGRP-induced relaxation of human coronary arteries is mainly mediated via the canonical CGRP receptor (CLR-RAMP1), while relaxation of human middle meningeal arteries can be mediated via both the canonical CGRP receptor and the adrenomedullin 1 receptor (CLR-RAMP2). Possibly, the balance between RAMP2 and RAMP1 expression in the meningeal vasculature, and thus the relative expression of the canonical CGRP receptor and adrenomedullin 1 receptor, could have implications for migraine susceptibility or treatment response. Future research should investigate whether RAMP2 predominance over RAMP1 in the meningeal vasculature indeed results in altered migraine susceptibility or results in a different response to anti-migraine medication in these patients. Moreover, the exact role of RCP in CGRP receptor signalling should be elucidated in future research.

## Electronic supplementary material

Below is the link to the electronic supplementary material.

Supplementary Material 1

## Data Availability

The data that support the findings of this study are available from the corresponding author upon reasonable request. Some data may not be made available because of privacy or ethical restrictions.
