# Supplementary material for: Differential expression of components of the CGRP-receptor family in human coronary and human middle meningeal arteries: functional implications
Source: J Headache Pain. 2024 Oct 10;25(1):176. doi: 10.1186/s10194-024-01863-7 (PMC11465939; doi:10.1186/s10194-024-01863-7)
Supplement: Supplementary file 1 — Supplementary Material 1 [file 10194_2024_1863_MOESM1_ESM.docx]

**Supplementary information**

| **Gene** | **Primer** | **Sequence** |
| --- | --- | --- |
| CLR | Forward | TTGTGCTGATTCCATGGCGA |
|  | Reverse | GGTAGAGACCAAAAGACCCTGG |
| CTR | Forward | CCCTGGAGACCTTCCAACAA |
|  | Reverse | CACGGTGGTTTGGACCTCA |
| RAMP1 | Forward | CCTCACCCAGTTCCAGGTAG |
|  | Reverse | TCCCTGTAGCTCCTGATGGT |
| RAMP2 | Forward | ACCAGGGTCAGAAGGGGG |
|  | Reverse | CTAATCATGGCCCAGTCGCA |
| RAMP3 | Forward | GCTCTGCGGTGGGTGT |
|  | Reverse | GCCCATCATGTCTGCGAAAG |
| RCP | Forward | GTGGAGATCCAGCTGATGGTG |
|  | Reverse | CAGAATGCTGGTGACGGTGT |
| Β-actin | Forward | CTCCCTGGAGAAGAGCTACG |
|  | Reverse | GAAGGAAGGCTGGAAGAGTG |
| GAPDH | Forward | TGCACCACCAACTGCTTAGC |
|  | Reverse | GGCATGGACTGTGGTCATGAG |
| HPRT1 | Forward | TGACACTGGCAAAACAATGCA |
|  | Reverse | GGTCCTTTTCACCAGCAAGCT |

***Table S1: Primers used for qPCR experiments***

**Figure S1: mRNA expression data and functional response in subpopulations.** *A) Relative expression of RAMP1 versus RAMP2 per tissue donor in human coronary artery and human middle meningeal artery. B) Distribution of RAMP1 and RAMP2 expression in human coronary artery and human middle meningeal artery. C) Relaxation to adrenomedullin in donors that showed a clear effect (≥ 1 log unit shift) of* AM_22-52_  *(n=4). D) Relaxation to adrenomedullin 2 in donors that showed a clear effect of* AM_22-52_  *(n=4). E) Relaxation to adrenomedullin in donors that did not show a clear effect (<1 log unit shift) of* AM_22-52_  *(n=5). F) Relaxation to adrenomedullin 2 in donors that did not show a clear effect of* AM_22-52_  *(n=6).*
